# Metaanalysis of the Performance of a Combined Treponemal and Nontreponemal Rapid Diagnostic Test for Syphilis and Yaws

**DOI:** 10.1093/cid/ciw348

**Published:** 2016-05-23

**Authors:** Michael Marks, Yue-Ping Yin, Xiang-Sheng Chen, Arnold Castro, Louise Causer, Rebecca Guy, Regina Wangnapi, Oriol Mitjà, Abdul Aziz, Rita Castro, Filomena da Luz Martins Pereira, Fasihah Taleo, Jérôme Guinard, Laurent Bélec, Ye Tun, Christian Bottomley, Ronald C. Ballard, David C.W. Mabey

**Affiliations:** 1 Clinical Research Department, Faculty of Infectious and Tropical Diseases, London School of Hygiene & Tropical Medicine; 2 Hospital for Tropical Diseases, University College London Hospitals NHS Trust, United Kingdom; 3 National Center for STD Control, China Center for Disease Control and Prevention, Beijing; 4 Chinese Academy of Medical Sciences Institute of Dermatology and Hospital of Skin Diseases, Nanjing, China; 5 Laboratory Reference & Research Branch, Division of STD Prevention, Centers for Disease Control and Prevention, Atlanta, Georgia; 6 Kirby Institute, University of New South Wales, Sydney, Australia; 7 Papua New Guinea Institute of Medical Research, Goroka, Eastern Highland Province; 8 Barcelona Institute for Global Health, Barcelona Centre for International Health Research, Hospital Clinic, University of Barcelona, Spain; 9 Lihir Medical Centre, International SOS, Newcrest Mining, Lihir Island, Papua New Guinea; 10 Ghana Health Services, Accra; 11 Unidade de Microbiologica Médica, Instituto de Higiene e Medicina Tropical, Lisbon, Portugal; 12 Ministry of Health, Port Vila, Vanuatu; 13 Laboratoire de Microbiologie, Centre Hospitalier Régional d'Orléans; 14 Laboratoire de Microbiologie, hôpital Européen Georges Pompidou, Assistance Publique-Hôpitaux de Paris; 15 Faculté de Médecine Paris Descartes, Université Paris Descartes (Paris V), Sorbonne Paris Cité, France; 16 Center for Global Health, Centers for Disease Control and Prevention, Atlanta, Georgia; 17 Department of Infectious Diseases Epidemiology, London School of Hygiene & Tropical Medicine, United Kingdom

**Keywords:** syphilis, yaws, sexually transmitted infections, point-of-care test, metaanalysis

## Abstract

***Background.*** The human treponematoses are important causes of disease. Mother-to-child transmission of syphilis remains a major cause of stillbirth and neonatal death. There are also almost 100 000 cases of endemic treponemal disease reported annually, predominantly yaws. Rapid diagnostic tests (RDTs) would improve access to screening for these diseases. Most RDTs cannot distinguish current and previous infection. The Dual Path Platform (DPP) Syphilis Screen & Confirm test includes both a treponemal (T1) and nontreponemal (T2) component and may improve the accuracy of diagnosis.

***Methods.*** We conducted a metaanalysis of published and unpublished evaluations of the DPP-RDT for the diagnosis of syphilis and yaws. We calculated the sensitivity, specificity, and overall agreement of the test compared with reference laboratory tests.

***Results.*** Nine evaluations, including 7267 tests, were included. Sensitivity was higher in patients with higher titer rapid plasma reagin (≥1:16) for both the T1 (98.2% vs 90.1%, *P* < .0001) and the T2 component (98.2% vs 80.6%, *P* < .0001). Overall agreement between the DPP test and reference serology was 85.2% (84.4%–86.1%). Agreement was highest for high-titer active infection and lowest for past infection.

***Conclusions.*** The RDT has good sensitivity and specificity of the treponemal and nontreponemal components both in cases of suspected syphilis and yaws, although the sensitivity is decreased at lower antibody titers.

## Abstract (precis)

A combined treponemal and nontreponemal rapid diagnostic test was found to have good sensitivity and specificity for both syphilis and yaws. The performance of both the treponemal and nontreponemal test components was strongly associated with the rapid plasma reagin titer.

The human treponematoses comprise venereal syphilis and the endemic treponematoses yaws, bejel, and pinta. Syphilis, caused by *Treponema pallidum* sp. *pallidum*, remains an important cause of both morbidity and mortality. The prevalence of syphilis is known to be particularly high among women attending ante-natal clinics in sub-Saharan Africa [1], and mother-to-child transmission of syphilis remains a major cause of stillbirth and neonatal death worldwide. It has been estimated that mother-to-child transmission of syphilis results in as many as 300 000 stillbirths and neonatal deaths each year in Africa alone [2]. These adverse pregnancy outcomes are entirely preventable through syphilis screening and appropriate treatment.

Yaws is an endemic treponemal infection caused by *T. pallidum* sp. *Pertenue* [3]. Although closely related to *T. pallidum* sp. *pallidum*, yaws is not sexually transmitted and predominantly affects children living in poor, rural humid communities in the tropics. Untreated yaws progresses to destructive lesions of the bones and soft tissues. Between 2008 and 2012 there were 300 000 cases of yaws reported to the World Health Organization (WHO). In 2012 the WHO launched a global effort to eradicate the disease by 2020 [4], and the development of a rapid diagnostic test (RDT) for yaws has been identified as a priority for the eradication program. As yaws is serologically indistinguishable from syphilis [5], tests developed for syphilis may also be of value in the diagnosis of yaws.

Diagnosis of treponemal infections is based on serological tests that are classified as treponemal specific, such as the *T. pallidum* particle agglutination assay (TPPA), *T. pallidum* hemagglutination assay (TPHA), enzyme-linked immunosorbent assay (ELISA), enzyme immunoassay (EIA), and fluorescent treponemal antibody test, or nontreponemal, such as the venereal disease research laboratory or the rapid plasma reagin (RPR) assay. Treponemal tests are highly specific but frequently remain positive for life following infection, regardless of treatment or natural clearance. Nontreponemal tests are less specific but reflect active disease more accurately, although positive nontreponemal results may also be seen in serofast patients. Diagnosis of treponemal infections is generally based on a combination of both types of test as well as clinical findings and history.

RDTs for treponemal infections are a relatively recent development; they allow wider access to diagnostic testing, particularly for communities where routine laboratory facilities are not available. RDTs facilitate improved screening, diagnosis, and treatment of syphilis in women presenting to antenatal clinics in low-resource settings [6] and reduce the morbidity and mortality associated with mother-to-child transmission of syphilis. For yaws, a RDT would be of value due to the low positive predictive value of clinical diagnosis alone [7, 8]. Validation and roll-out of a RDT would lead to improved epidemiological data on yaws worldwide, which is a priority in facilitating eradication [4, 9].

A major limitation to most treponemal RDTs is that they are based on detection of treponemal-specific antibodies and therefore cannot distinguish between current and past infection. Resulting false positives lead to overtreatment of syphilis, as well as problems in interpreting epidemiological data for both syphilis and yaws. The Dual Path Platform (DPP-RDT) Syphilis Screen & Confirm test kit (Chembio, Medford, New York) is the first commercial RDT to give both a “treponemal” result and a “nontreponemal” result [10]. Therefore, it can assist in distinguishing between current and past infection, which may make it a more useful test in clinical practice. The kit is a lateral flow assay that detects both immunoglobulin (Ig)M and IgG antibodies against a recombinant *T. pallidum* antigen and a nontreponemal antigen. Several recent publications have reported on the performance of this assay, noting good test performance; however, variations have been noted in the sensitivity and specificity of the nontreponemal component of the test in particular. To provide more accurate estimates of performance, we conducted an individual patient-level metaanalysis on the performance of the DPP-RDT for the diagnosis of both syphilis and yaws.

## METHODS

For this review and metaanalysis we used the Preferred Reporting Items for Systematic Reviews and Meta Analyses guidelines [11].

### Search Strategy

We searched PubMed from 1 January 1960 to 1 August 2015 using the terms “RDT” OR “point of care test” AND “syphilis” OR “yaws” (and variations). We searched reference lists of identified articles and contacted individuals and research groups known to have undertaken unpublished evaluation studies to identify other relevant datasets.

#### Inclusion Criteria

An article was included if it evaluated the sensitivity and specificity of the Chembio Syphilis Screen & Confirm Rapid Diagnostic Test to detect syphilis or yaws. Laboratory, clinic, and field-based studies that sampled a consecutive series of patients or randomly selected series of patients were eligible. Non-English language publications were eligible for inclusion.

#### Exclusion Criteria

Articles were excluded if they did not contain primary data (eg, editorials, reviews, and commentaries) or referred to conference proceedings and were not accompanied by a full description of the research.

### Data Extraction and Management

The first author screened all titles and abstracts, and the full text was obtained for any potentially relevant articles. Full-text articles were reviewed to determine whether they met the inclusion criteria; where this was uncertain, articles were reviewed by a coauthor and disagreements were resolved by discussion. Data were initially extracted by the first author and double-checked by the coauthors.

For each study we extracted data on the sample type used for the DPP-RDT and the reference treponemal and nontreponemal assay used. For each individual patient we recorded data on the presence and stage of clinical disease, the result of the reference treponemal and nontreponemal assays, and the result of the DPP-RDT.

Sample types were classified as serum, plasma, whole blood, or finger-prick. RDT results are dichotomous; reference RPR results were deemed positive at a titer ≥1:1; an RPR titer ≥1:16 was considered a high-titer RPR.

### Statistical Analyses

We estimated the sensitivity and specificity of the DPP-RDT by comparing the performance of the DPP treponemal result (T1 component) to reference treponemal serology and by comparing the performance of the DPP non treponemal result (T2 component) to reference nontreponemal serology. Exact confidence intervals were calculated for each of these estimates using the binomial distribution. The inverse variance was used to weight each study in the metaanalysis. We calculated the *I*^2^ statistic to quantify study variability, and sensitivities and specificities were compared between prespecified subgroups including the RPR titer, disease, sample type, and clinical disease stage. We conducted multivariable logistic regression to assess test variables including disease, RPR titer, sample type, and reference treponemal test that were significantly associated with test performance.

We assessed the overall performance of the DPP-RDT in detecting categories of infection. We classified the outcome of reference serology as active infection (treponemal and nontreponemal test positive), past infection (treponemal test positive and nontreponemal test negative), and no history of infection (both tests negative). We classified the outcome of the DPP-RDT correspondingly, as defining active infection (both elements positive), past infection (only the treponemal element positive), and no previous infection (both negative). We calculated the overall agreement between the outcome of the DPP-RDT and the reference serology. All analyses were conducted using Stata 13.1 (StataCorp, College Station, Texas).

The ethics committee of the London School of Hygiene & Tropical Medicine approved the study (ref. 8908).

## RESULTS

**Figure 1.**
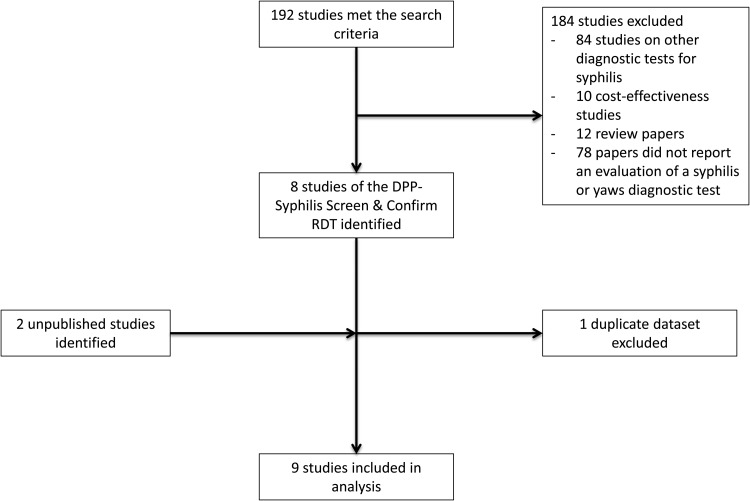
Search results. Abbreviations: DPP, Dual Path Platform; RDT, rapid diagnostic test.

A total of 192 studies met the search criteria, and 10 articles met the inclusion criteria [10, 12–18]. Of these, 2 articles reported data on the same set of patients; 1 article evaluated the treponemal component of 4 RDTs including the DPP-RDT [18], the second study evaluated both the treponemal and nontreponemal component of the DPP-RDT and was included in this analysis [13]. Two unpublished studies were identified by contacting groups known to have undertaken evaluations of the DPP-RDT (Figure 1).

Overall, individual-level data from 9 studies involving 7267 test results were included in the metaanalysis. In all included studies the individual conducting the RDT was reported to be blinded to the results of the reference serological tests. The countries and characteristics of the included studies are shown in Table 1. In 1 study [10] individual-level data were only available for 61.8% of the full dataset. The performance of the DPP test did not differ between the full dataset and the individual-level data that were available (data not shown).

**Table 1.** Study Characteristics

Study, First Author	Study Site	Disease	Reference Treponemal Test	Reference Nontreponemal Test	Sample Type	Sample Size	Year of Publication
Ayove [16]	Papua New Guinea	Yaws	TPHA	RPR	Finger-prick	199	2014
					Plasma	504	
Aziz	Ghana	Yaws	TPPA	RPR	Finger-prick	255	Unpublished data
Castro [10]	United States	Syphilis	TPPA	RPR	Serum	1168^a^	2010
R Castro [12]	Portugal	Syphilis	TPHA	RPR	Serum	248	2014
Causer [13]	Australia	Syphilis	ELISA/EIA	RPR	Serum	1005	2015
Guinard [17]	France	Syphilis	ELISA/EIA	RPR	Serum	100	2013
Marks [15]	Solomon Islands	Yaws	TPPA	RPR	Serum	415	2014
Taleo	Vanuatu	Yaws	TPPA	RPR	Finger-prick	238	Unpublished data
Yin [14]	China	Syphilis	TPPA	TRUST	Plasma	1323	2013
					Whole blood	1324	
					Finger-prick	488	

Abbreviations: EIA, enzyme immunoassay; ELISA, enzyme-linked immunosorbent assay; RPR, rapid plasma reagin; TPHA, *Treponema* *pallidum* hemagglutination assay; TPPA, *Treponema* *pallidum* particle agglutination assay; TRUST, toluidine red unheated serum test.

^a^ The published study size was 1889; however, individual-level data was only available for 1168 tests.

Of patients included in the study, 5656 (77.8%) underwent treponemal testing for suspected syphilis and 1611 for suspected yaws. Individual-level clinical data on the presence or absence of disease status were available for 2636 patients (36.3%). Of patients for whom clinical data were available, 1417 (53.8%) had clinical evidence of either active syphilis or active yaws at the time of testing and 1219 (46.2%) had no clinical evidence of active disease. The reference treponemal test was positive in 4075 individuals (56.1%) and the reference nontreponemal test was positive in 3112 (42.8%).

**Figure 2.**
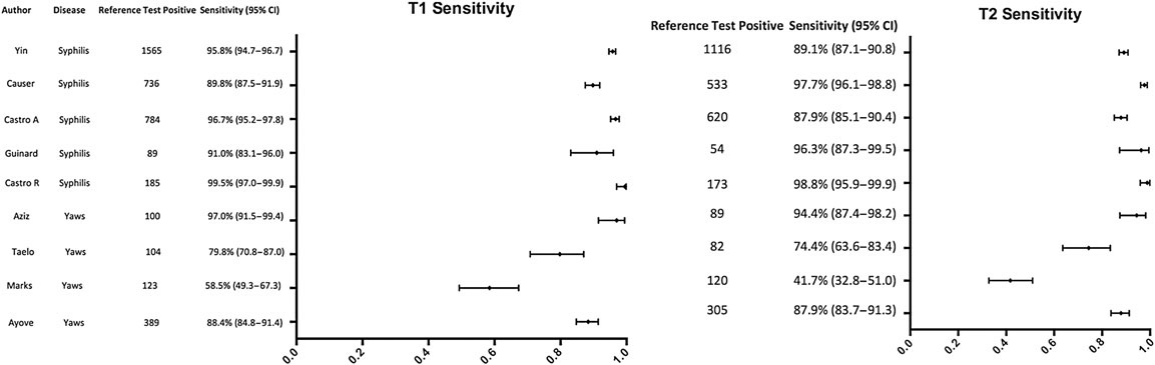
Forest plot of the sensitivity of the T1 (treponemal) and T2 (non-treponemal) components in comparison to reference treponemal and nontreponemal assays. Abbreviation: CI, confidence interval.

**Figure 3.**
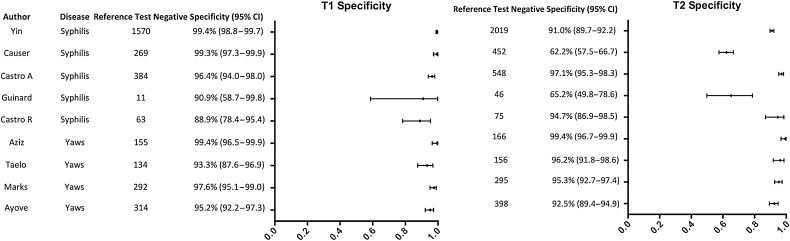
Forest plot of specificity of the T1 (treponemal) and T2 (non-treponemal) components in comparison to reference treponemal and nontreponemal assays. Abbreviation: CI, confidence interval.

There was significant heterogeneity across the studies in the sensitivity of both the treponemal (*I*^2^ 95.70%, *P* < .01) and nontreponemal components (*I*^2^ 96.70%, *P* < .01) (Figure 2). When the results were restricted to high-titer samples, the heterogeneity was no longer statistically significant for either treponemal (*I*^2^ 26.13%, *P* = .21) or nontreponemal (*I*^2^ 26.13%, *P* = .79) components (Supplementary Figure 1). There was also heterogeneity in the specificity of the treponemal component (*I*^2^ 78.98%, *P* < .01) and the nontreponemal component (*I*^2^ 97.59%, *P* < .01; Figure 3). As there was significant heterogeneity between studies, an overall pooled summary estimate of the sensitivity and specificity of the RDT across the full dataset is not reported. Sensitivity was higher for specimens from patients with a high-titer RPR (≥1:16) (n = 1351) compared with specimens with a lower RPR titer (<1:16) for both the treponemal component (98.2% vs 90.1%, *P* < .0001) and the nontreponemal component (98.2% vs 80.6%, *P* < .0001; Table 2). If all RPR-positive samples are considered positive, the specificity of the treponemal component was 98.0% and the specificity of the nontreponemal component was 89.4%. If only samples with a high-titer RPR (≥1:16) are considered positive, the specificity of the treponemal component was 91.2% (Table 2).

**Table 2.** Overall Sensitivity and Specificity of the Dual Path Platform Rapid Diagnostic Test by Rapid Plasma Reagin Titer

RPR Titer	n	Reference Test Positive	DPP Positive	Sensitivity (95% CI)	Reference Test Negative	DPP Negative	Specificity (95% CI)
RPR <1:16							
Treponemal test	5916	2736	2464	90.1% (88.9–91.2)	3180	3115	98.0% (97.4–98.4)
Nontreponemal test	5916	1761	1419	80.6% (78.7–82.4)	4155	3714	89.4% (88.4–90.3
RPR ≥1:16							
Treponemal test	1351	1339	1315	98.2% (97.3–98.8)	12	11	91.2% (61.5–99.8)
Nontreponemal test	1351	1351	1327	98.2% (97.4–98.9)			

Abbreviations: CI, confidence interval; DPP, Dual Path Platform; RPR, rapid plasma reagin.

The sensitivities of both test components were higher in patients with syphilis than in patients with yaws at low titers but not at high titers. For high-titer specimens the sensitivity of the treponemal component was 98.4% for syphilis and 97.6% for yaws, and the sensitivity of the nontreponemal components was 98.7% for syphilis and 96.6%for yaws. For low-titer specimens the sensitivity of the treponemal component was 93.1% for syphilis compared with 73.5% for yaws and the sensitivity of the nontreponemal component was 85.0% for syphilis compared with 59.1% yaws (*P* < .0001 in both cases). The specificity of the treponemal component was slightly higher in patients tested for syphilis compared with those tested for yaws for low-titer specimens, while the converse was true for the nontreponemal component (Table 3).

**Table 3.** Sensitivity and Specificity of the Dual Path Platform Rapid Diagnostic Test Stratified by Disease and Rapid Plasma Reagin Titer

Disease	n	Reference Test Positive	DPP Positive	Sensitivity (95% CI)	Reference Test Negative	DPP Negative	Specificity (95% CI)
*Syphilis*							
RPR <1:16							
Treponemal test	4600	2310	2151	93.1% (92.0–94.1)	2290	2256	98.5% (97.9–99.0)
Nontreponemal test	4600	1460	1241	85.0% (83.1–86.8)	3140	2750	87.6% (86.4–88.7)
RPR ≥1:16							
Treponemal test	1056	1049	1032	98.4% (97.4–99.1)	7	7	100% (59–100)
Nontreponemal test	1056	1056	1042	98.7% (97.8–99.3)			
*Yaws*							
RPR <1:16							
Treponemal test	1316	426	313	73.5% (69.0–77.6)	890	859	96.5% (95.1–97.6)
Nontreponemal test	1316	301	178	59.1% (53.3–64.7)	1015	964	95.0% (93.4–96.2)
RPR ≥1:16							
Treponemal test	295	290	283	97.6% (95.1–99.0)	5	4	80.0% (28.4–99.5)
Nontreponemal test	295	295	285	96.6% (93.8–98.4)			

Abbreviations: CI, confidence interval; DPP, Dual Path Platform; RPR, rapid plasma reagin.

There were only minor differences in the performance of the test based on the specimen type used (Supplementary Table 1). The sensitivity and specificity of the treponemal component varied depending on the reference treponemal assay used (Supplementary Table 2). Compared with use of TPPA as the reference treponemal test, the sensitivity of the treponemal component was lower when the reference test was ELISA (*P* < .001) and the specificity was lower when TPHA was taken as the reference standard. The sensitivity of both the treponemal and nontreponemal components was higher in individuals with evidence of clinical disease than in asymptomatic cases (Supplementary Table 3).

In multivariable logistic regression a higher RPR titer was significantly associated with an increased sensitivity of both the treponemal and the nontreponemal components (*P* < .001) after controlling for other variables. After controlling for other variables, the sensitivity was lower for both test components in individuals being tested for yaws than in those tested for syphilis and when the test was performed on serum (*P* < .001 in both cases). After controlling for other variables, the specificity of the treponemal component was higher when the reference standard was either TPPA or TPHA compared with EIA or ELISA (*P* < .0001 in both cases). The specificity of the nontreponemal component was significantly associated with the RPR titer after controlling for other variables (*P* < .023).

Overall agreement between the DPP test and reference serology was 85.2% (84.4%–86.1%). Agreement was highest for high-titer active infection and lowest for past infection (Table 4). The lack of agreement in this group was due to misclassification of both treponemal components and nontreponemal components (data not shown).

**Table 4.** Agreement of the Dual Path Platform Test Kit Classification Relative to Reference Serology

Serological Classification	Reference Test Classification	Dual Path Platform Test Classification	Agreement (95% Confidence Interval)
Active	3021	2668	88.3% (87.1–89.4)
Active (high titer ≥1:16)	1339	1306	97.5% (96.6–98.3)
Active (low titer <1:16)	1682	1362	81.0% (79.1–82.8)
Past infection	1054	570	54.1% (51.1–57.1)
No infection	3101	2938	94.7% (93.9–95.6)
False-positive rapid plasma reagin^a^	91	19	20.9% (13.1–30.7)

^a^ A rapid plasma reagin (RPR) was considered a false positive if the treponemal test was negative. This may be overly conservative as the RPR may become positive before the treponemal test. This definition would not affect our estimate of the agreement between the Dual Path Platform test and the reference test in this scenario.

## DISCUSSION

In this metaanalysis we combined data collected from more than 7200 patients to evaluate the performance of the combined treponemal–nontreponemal RDT for the diagnosis of syphilis and yaws. The use of individual patient data from a large number of samples allowed us to explore which factors are independently associated with test performance. We found that the DPP Syphilis Screen & Confirm RDT has good sensitivity and specificity compared with reference serology in cases of both suspected syphilis and yaws. As previously reported in one study, the sensitivity of the nontreponemal component of the RDT is related to the patient's RPR titer [15], with significantly higher sensitivity of the DPP kit for high-titer RPRs. We demonstrated that the sensitivity of the treponemal component is also related to the RPR titer. This finding was true even after controlling for other relevant variables such as the sample type, disease, and reference test used. As a result, the DPP-RDT showed excellent overall sensitivity for high-titer infections (RPR ≥1:16; 97.5%) but lower sensitivity for low-titer active infections (81.0%). Although treponemal tests are commonly reported as either positive or negative, quantitative testing is possible and titers to certain treponemal antigens decline following treatment [19]. This, it is likely that these findings reflect an overall reduced sensitivity of the RDT at lower antibody titers. As a result, some low-titer positive patients may be missed when the DPP-RDT is used. As the DPP-RDT is designed to be used as a point-of-care test, the lower sensitivity noted when testing serum samples may be of less clinical relevance. However, clinicians should be aware of this when considering how to roll out rapid diagnostic testing.

Improved screening of women attending antenatal care for syphilis is a priority intervention to reduce the mortality and morbidity associated with mother-to-child transmission of syphilis. Most adverse pregnancy outcomes due to syphilis are seen in mothers with an RPR titer ≥1:8 [20], and our findings confirm that the DPP-RDT has a high sensitivity in this group (97.6%). Adoption of the DPP assay as the basis for treatment decision would therefore be likely to detect a high proportion of active infections. Conversely, it would reduce the number of women having unnecessary treatment, as around half of those with past treated infection would show up as negative on the test compared with positive on a standard treponemal-only RDT.

This study was not designed to assess the cost-effectiveness of a combined treponemal and nontreponemal RDT, but other studies have shown that this is highly dependent on both the prevalence of disease and the cost of the RDT [21]. A number of RDTs used for antenatal settings now combine a treponemal test with a human immunodeficiency virus ( HIV) test [22]. Any decision regarding the correct RDT selection and testing strategy is likely to vary between countries, depending on the prevalence of syphilis, yaws, and HIV and the cost and availability of the RDTs.

Clinical diagnosis of yaws alone does not have a high predictive value, and the addition of a RDT would be a significant advantage. In this metaanalysis, the DPP-RDT performed better in patients with suspected syphilis than in those with suspected yaws. This finding was explained predominantly by the lower sensitivity in yaws patients with low titers. Although lower titers are often found in patients with yaws compared with those with syphilis [23], it is unclear why the test performance should be worse in patients with yaws when controlling for antibody titer. These findings suggest that the DPP-RDT may be adequate in pre-mass treatment campaigns when there are many active cases with high-titer disease but that, as the number of active cases declines, it may be necessary to use a different testing strategy, either repeated testing or adoption of an alternative test with a higher sensitivity for low-titer disease.

A limitation of our study was that the full individual clinical dataset was not available for 1 study [10]. The missing data represent less than 10% of the total set included in this study, and the reported results from the full dataset do not differ significantly from the subset used in this metaanalysis. Consequently, it seems unlikely that the missing data would substantially alter our findings. All data analyzed were collected as part of research studies and it is recognized that test performance may not be as good in a real-world setting in the hands of the end users (healthcare workers) and that the use of test results may be influenced by factors other than simply the result [24]. Training and support for healthcare workers has been shown to significantly improve use of RDTs in other areas [25] and should be a key component of the roll-out of RDTs for syphilis and yaws.

This metaanalysis demonstrates that the DPP-RDT has high sensitivity and specificity for both treponemal and nontreponemal antibodies. Our analysis includes a large number of patients from many countries who were enrolled in studies and tested for syphilis or yaws. Our large sample size allows us to provide the most accurate estimates published to date of the test performance across a range of subgroups. The major limitation of the DPP-RDT is its reduced sensitivity for low-titer disease. As RPR titers tend to be higher in patients with syphilis than in patients with yaws, this reduced sensitivity is likely to be a greater problem when using the test as part of yaws eradication efforts, especially as a high-sensitivity assay will be needed to ensure all cases are detected to confirm the final eradication status of the infection. Combined treponemal–nontreponemal assays offer a number of advantages over treponemal only RDTs. Our data provide evidence to support the decision to use the DPP-RDT as one such assay.

## Supplementary Data

Supplementary materials are available at http://cid.oxfordjournals.org. Consisting of data provided by the author to benefit the reader, the posted materials are not copyedited and are the sole responsibility of the author, so questions or comments should be addressed to the author.

Supplementary Data
