# Supplementary material for: Metaanalysis of the Performance of a Combined Treponemal and Nontreponemal Rapid Diagnostic Test for Syphilis and Yaws
Source: Clin Infect Dis. 2016 May 23;63(5):627–33. doi: 10.1093/cid/ciw348 (PMC4981758; doi:10.1093/cid/ciw348)
Supplement: Supplementary Data [file supp_63_5_627__index.html]

Metaanalysis of the Performance of a Combined Treponemal and Nontreponemal Rapid Diagnostic Test for Syphilis and Yaws — Supplementary Data 

# Metaanalysis of the Performance of a Combined Treponemal and Nontreponemal Rapid Diagnostic Test for Syphilis and Yaws

## Supplementary Data

Supplementary Data

- Supplementary Figure 1 - pdf file
- Supplementary Tables - docx file
