# Supplementary material for: Metaanalysis of the Performance of a Combined Treponemal and Nontreponemal Rapid Diagnostic Test for Syphilis and Yaws
Source: Clin Infect Dis. 2016 May 23;63(5):627–33. doi: 10.1093/cid/ciw348 (PMC4981758; doi:10.1093/cid/ciw348)

Supplementary Figure 1: Forest plot of specificity of the T1 (treponemal) and T2 (non-treponemal) components in comparison to reference treponemal and nontreponemal assays. Abbreviation: CI, confidence interval.

Author	Disease	Reference Test Positive	Sensitivity (95% CI)	Reference Test Positive	Sensitivity (95% CI)
Yin	Syphilis	330	99.7% (98.3 - 99.9)	337	97.6% (95.4 - 98.9)
Causer	Syphilis	335	95.5% (92.7 - 97.5)	335	98.2% (96.1 - 99.3)
Castro A	Syphilis	221	99.5% (97.5 - 99.9)	221	100% (98.3 - 100)
Guinard	Syphilis	29	100% (88.0 - 100)	29	100% (88.1 - 100)
Castro R	Syphilis	134	100% (97.2 - 100)	134	100% (97.3 - 100)
Aziz	Yaws	65	100% (94.4 - 100)	65	96.7% (89.3 - 99.6)
Taelo	Yaws	38	92.1% (78.6 - 98.3)	38	94.7% (82.3 - 99.4)
Marks	Yaws	25	92.0% (73.9 - 99.0)	25	92.0% (73.9 - 99.0)
Ayove	Yaws	162	98.8% (95.6 - 99.9)	167	97.6% (93.9 - 99.3)

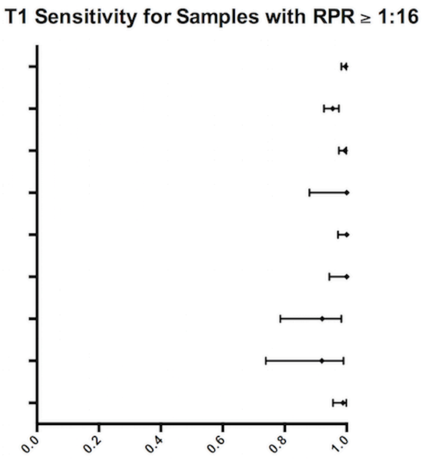

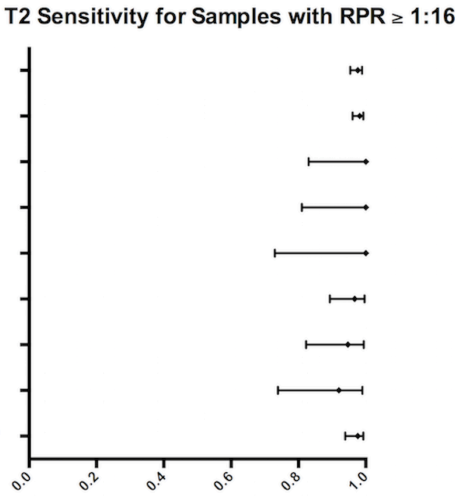

Supplement: Supplementary Data [file supp_ciw348_ciw348supp_fig1.pdf]
