# Supplementary material for: Metaanalysis of the Performance of a Combined Treponemal and Nontreponemal Rapid Diagnostic Test for Syphilis and Yaws
Source: Clin Infect Dis. 2016 May 23;63(5):627–33. doi: 10.1093/cid/ciw348 (PMC4981758; doi:10.1093/cid/ciw348)
Supplement: Supplementary Data [file supp_ciw348_ciw348supp_table.docx]

| **Supplementary Table 1** | **Sensitivity and Specificity Stratified by Sample Type and RPR Titre** | | | | | |  | |  | |
| --- | --- | --- | --- | --- | --- | --- | --- | --- | --- | --- |
| **By Sample Type** | **n** | **Reference Test Positive** | **DPP Positive** | **Sensitivity (95% CI)** | **Reference Test Negative** | **DPP Negative** | | **Specificity (95% CI)** | |  |
| *Finger-Prick* |  |  |  |  |  |  | |  | |  |
| RPR <1:16 |  |  |  |  |  |  | |  | |  |
| Treponemal Test | 965 | 359 | 321 | 89.4% (85.6-92.4%0 | 606 | 591 | | 97.5% (96.0-98.6%) | |  |
| Non-Treponemal Test | 965 | 227 | 174 | 76.7% (80.6-82.0%) | 738 | 689 | | 93.4% (91.3-95.0%) | |  |
|  |  |  |  |  |  |  | |  | |  |
| RPR ≥1:16 |  |  |  |  |  |  | |  | |  |
| Treponemal Test | 215 | 214 | 210 | 98.1% (95.3-99.5%) | 1 | 1 | | 100% (25-100%) | |  |
| Non-Treponemal Test | 215 | 215 | 208 | 96.7% (93.4-98.7%) |  |  | |  | |  |
|  |  |  |  |  |  |  | |  | |  |
| *Plasma* |  |  |  |  |  |  | |  | |  |
| RPR <1:16 |  |  |  |  |  |  | |  | |  |
| Treponemal Test | 1575 | 696 | 626 | 89.9% (87.5-92.1%) | 879 | 865 | | 98.4% (97.3-99.1%) | |  |
| Non-Treponemal Test | 1575 | 439 | 374 | 85.2% (81.5-88.4%) | 1136 | 1043 | | 93.0% (90.1-93.3%) | |  |
|  |  |  |  |  |  |  | |  | |  |
| RPR ≥1:16 |  |  |  |  |  |  | |  | |  |
| Treponemal Test | 253 | 245 | 243 | 99.2% (97.1-99.9%) | 8 | 7 | | 87.5% (47.3-99.7%) | |  |
| Non-Treponemal Test | 253 | 253 | 246 | 97.2% (94.4-98.9%) |  |  | |  | |  |
|  |  |  |  |  |  |  | |  | |  |
| *Whole Blood* |  |  |  |  |  |  | |  | |  |
| RPR <1:16 |  |  |  |  |  |  | |  | |  |
| Treponemal Test | 1184 | 508 | 487 | 95.9% (93.8-07.4%) | 676 | 671 | | 99.3% (98.3-99.8%) | |  |
| Non-Treponemal Test | 1184 | 319 | 268 | 84.0% (79.5-87.9%) | 865 | 787 | | 91.0% (88.9-92.8%) | |  |
|  |  |  |  |  |  |  | |  | |  |
| RPR ≥1:16 |  |  |  |  |  |  | |  | |  |
| Treponemal Test | 139 | 136 | 136 | 100% (97.3-100%) | 3 | 3 | | 100% (29.2-100%) | |  |
| Non-Treponemal Test | 139 | 139 | 137 | 98.6% (94.9-99.8%) |  |  | |  | |  |
|  |  |  |  |  |  |  | |  | |  |
| *Serum* |  |  |  |  |  |  | |  | |  |
| RPR <1:16 |  |  |  |  |  |  | |  | |  |
| Treponemal Test | 2192 | 1173 | 1030 | 87.8% (85.8-89.6%) | 1019 | 988 | | 97.0% (95.7-97.9%) | |  |
| Non-Treponemal Test | 2192 | 776 | 603 | 77.7% (74.6-80.6%) | 1416 | 1195 | | 84.4% (82.4-86.2%) | |  |
|  |  |  |  |  |  |  | |  | |  |
| RPR ≥1:16 |  |  |  |  |  |  | |  | |  |
| Treponemal Test | 744 | 744 | 726 | 97.6% (96.2-98.6%) |  |  | |  | |  |
| Non-Treponemal Test | 744 | 744 | 736 | 98.9% (97.9-99.5%) |  |  | |  | |  |

| **Supplementary Table 2** | **Performance of the Treponemal Test Component by Reference Standard** | | | | | |  |
| --- | --- | --- | --- | --- | --- | --- | --- |
|  | **n** | **Reference Test Positive** | **DPP Positive** | **Sensitivity (95% CI)** | **Reference Test Negative** | **DPP Negative** | **Specificity (95% CI)** |
| Overall | 7267 | 4075 | 3779 | 92.7% (91.9-93.5%) | 3192 | 3126 | 97.9% (97.4-98.4) |
| EIA | 1005 | 736 | 661 | 89.8% (87.3-91.9%) | 269 | 267 | 99.2% (97.3-99.9%) |
| ELISA | 100 | 89 | 81 | 91.0% (83.1-96.0%) | 11 | 10 | 90.9% (58.7-99.8%) |
| TPHA | 951 | 574 | 528 | 92.0% (89.5-94.1%) | 377 | 355 | 94.1% (91.3-96.3%) |
| TPPA | 5211 | 2676 | 2509 | 95.3 (04.4-96.1%) | 2535 | 2494 | 98.4% (97.8-98.9%) |

| **Supplementary Table 3** | **Test Performance Stratified by Disease and Clinical Stage** | | | |  |  |  | |  | |
| --- | --- | --- | --- | --- | --- | --- | --- | --- | --- | --- |
| **By Clinical Stage*** | **n** | **Reference Test Positive** | **DPP Positive** | **Sensitivity (95% CI)** | **Reference Test Negative** | **DPP Negative** | | **Specificity (95% CI)** | |  |
| **Syphilis** |  |  |  |  |  |  | |  | |  |
| *Asymptomatic* | 785 |  |  |  |  |  | |  | |  |
| Treponemal Test |  | 535 | 488 | 91.2% (88.5-93.5%) | 250 | 242 | | 95.6% (93.8-98.6%) | |  |
| Non-Treponemal Test |  | 378 | 358 | 94.7% (91.9 - 96.7%) | 407 | 273 | | 67.1% (62.3 - 71.6%) | |  |
|  |  |  |  |  |  |  | |  | |  |
| *Primary Disease* | 125 |  |  |  |  |  | |  | |  |
| Treponemal Test |  | 111 | 104 | 88.8% (81.9 - 93.7%) | 14 | 12 | | 85.7% (57.2 - 98.2%) | |  |
| Non-Treponemal Test |  | 105 | 101 |  | 20 | 12 | |  | |  |
|  |  |  |  |  |  |  | |  | |  |
| *Secondary Disease* |  |  |  |  |  |  | |  | |  |
| Treponemal Test | 115 | 115 | 111 | 96.5% (91.3 - 99.0%) | N/A | N/A | | N/A | |  |
| Non-Treponemal Test |  | 114 | 110 | 96.2% (90.5 - 98.9%) | 1 | 0 | | N/A | |  |
|  |  |  |  |  |  |  | |  | |  |
|  |  |  |  |  |  |  | |  | |  |
| **Yaws** |  |  |  |  |  |  | |  | |  |
| *Asymptomatic* | 434 |  |  |  |  |  | |  | |  |
| Treponemal Test |  | 128 | 77 | 60.2% (51.1 - 68.7%) | 306 | 299 | | 97.7% (95.3 - 99.1%0 | |  |
| Non-Treponemal Test |  | 123 | 53 | 43.1% (34.2 - 52.3%) | 311 | 297 | | 95.5 - 92.6%) | |  |
|  |  |  |  |  |  |  | |  | |  |
| *Primary Disease* | 878 |  |  |  |  |  | |  | |  |
| Treponemal Test |  | 441 | 385 | 87.3% (83.8 - 90.3%) | 437 | 419 | | 95.9% (93.6 - 97.5%) | |  |
| Non-Treponemal Test |  | 355 | 312 | 87.9% (84.0 - 91.1%) | 523 | 494 | | 94.5% (92.1 - 96.3%) | |  |
|  |  |  |  |  |  |  | |  | |  |
| *Secondary Disease* | 299 |  |  |  |  |  | |  | |  |
| Treponemal Test |  | 147 | 134 | 91.2% (85.4 - 95.2%) | 152 | 145 | | 95.4% (90.7 - 98.1%) | |  |
| Non-Treponemal Test |  | 118 | 98 | 83.1% (75.0 - 89.3%) | 181 | 173 | | 95.6% (91.5 - 98.1%) | |  |

*Information on the clinical status of the patient was available for 2636 (36.3%) of samples
